# Dietary exposure of radionuclides and heavy metals in adult residents in a high background natural radiation area using duplicate diet method

**DOI:** 10.1038/s41598-022-19979-8

**Published:** 2022-10-06

**Authors:** Baolu Yang, Fei Tuo, Qiang Zhou, Jing Zhang, Zeshu Li, Chaoya Pang

**Affiliations:** Key Laboratory of Radiological Protection and Nuclear Emergency, National Institute for Radiological Protection, Chinese Center for Disease Control and Prevention, Beijing, 100088 China

**Keywords:** Environmental impact, Chemical safety, Environmental monitoring

## Abstract

Intake of radionuclides and heavy metals through food consumption is one of the important pathways for long-term health considerations. In this paper, the dietary exposure to radionuclides (^210^Pb, ^210^Po, ^226^Ra, ^228^Ra, ^40^K, ^137^Cs and ^129^I) and heavy metals (As, Hg, Pb, Cd and U) of adult residents in the high background natural radiation area (HBNRA) in Yangjiang, China, was comprehensively assessed using duplicate diet method. The estimated effective dose received by the inhabitants in HBNRA from ingestion of radionuclides was 0.33 mSv/y, and the associated lifetime cancer risk was 1.1 × 10^–3^. Both the dose and cancer risk to humans were at the acceptable range, and showed no difference between the HBNRA and the control area. With respect to heavy metals, the estimated daily intake of heavy metals (DIM) values for As, Hg, Pb, Cd and U in HBNRA were 0.47, 0.03, 15.0, 0.26 and 0.04 μg/kg bw/d, respectively, and the corresponding target hazard quotient (THQ) were 1.58, 0.09, 3.7, 2.56, 0.18. The DIM and THQ of Cd and U in HBNRA were similar to the control area, but the DIM and THQ of Pb were much higher than the corresponding values of 0.39 and 0.03 in the control area. The hazard index (HI) value of heavy metals in HBNRA was almost twice that of the control area. This suggests that the inhabitants in the HBNRA may have a health risk associated with the heavy metals.

## Introduction

The world's high background natural radiation areas (HBNRA), such as Kerala (India), Guarapari (Brazil), Ramsar (Iran), and Yangjiang (China), have received more attention in recent years because the radiation dose received by the inhabitants is higher than the normal background radiation areas by one to two orders, and a few HBNRA are even above the annual effective dose limit of 20 mSv for radiation workers^1,2^. Considering the linear no-threshold (LNT) model, namely, the dose–response for cancer and hereditary effects has a simple proportionate relationship between dose and risk at low dose level, the public living in HBNRA may be at a high risk for developing cancer and hereditary effects. Although the existing epidemiological studies have not provide strong evidence of health effects associated with the effect of low doses of ionizing radiation, it is necessary to make a comprehensive assessment of the existing exposure situation in HBNRA to get accurate radiation dose data to facilitate the epidemiology studies of populations living in HBNRA.

Ingestion of radionuclides through food is one of the important pathways for internal radiation exposure, accounting for a large part of radiation doses of residents. Due to higher levels of natural radionuclides, the inhabitants of HBNRA may receive higher internal exposure doses through their food than residents from normal background radiation area^3^. In fact, higher activity concentrations of ^238^U, ^226^Ra, ^228^Ra, ^210^Pb, ^228^Th and ^40^K in HBNRA local foods have been reported worldwide, such as Poços de Caldas, Brazil^3,4^, southwest HBNRA, Cameroon^5^, Kanyakumari, India^6,7^, Ramsar, Iran^8^ and Yangjiang, China^9^. However, the radioactive content of a specific food item does not necessarily reflect the dose ingested by resident, owing to the effect of kitchen preparation and the variety of available food items. Therefore, it is necessary to assess the dietary exposure based on the complete meals consumed.

In addition to natural radionuclides, the ingested dose may also arise from the artificial radionuclides released into the environment as a result of atmospheric nuclear weapons tests and nuclear power plant accidents. ^137^Cs (*t*_1/2_ = 30 years) and ^129^I (*t*_1/2_ = 1.57 × 10^7^ years) are one of the major artificial radionuclides which are recognized as a persistent environmental pollutant due to their long half-lives. It is well known that ^137^Cs is one of the relatively few artificial radionuclides which are of radiological concern being a nuclide which caused and still causes food contamination. In contrast to ^137^Cs, ^129^I has received less radiological attention in food probably because of analytical difficulties. However, ^129^I is accumulated in various human tissues because of the high bioavailability of iodine^10^. Dogru et al.^11^ reported that the ^129^I radioactivity level in the water supply may increase the risk of the nodular formation of the thyroid tissue in the eastern part of Turkey. Therefore, its concentration in the HBNRA local foods should be evaluated for assessing the dietary exposure doses for inhabitants.

In fact, environmental pollutants including radiation seldom occur in isolation. Areas with high levels of natural radionuclides in soils have been reported to be accompanied by significant heavy metal levels^12,13^. Because soil is the source of nutrients for crops, heavy metals in the soils may be transferred to different foods through the absorption of crops. Just as radionuclides can cause human exposure to radiation through food ingestion, ingestion of food contaminated with heavy metals can also have chemical toxic effects on our bodies. However, few data are available in the literature regarding the levels of heavy metal in food of HBNRA. Consequently, the levels of the heavy metals in food of HBNRA are a cause for concern and should be studied further.

There are three methods for evaluating dietary chemical pollutants and the nutrient intake of residents in a country or region: single-food selectivity study, total diet study and duplicate diet method^14,15^. Single-food selectivity study focuses on single food rather than the entire population and is often used for initial assessment. Total diet study is considered as the most suitable method for estimating a country or a large population area, but this method lacks the accuracy of actual dietary exposure measurements and does not capture the important contributions of resident storage, preparation and consumption^15,16^. Although duplicate diet method is difficult to carry out on a large scale due to its large workload, it is considered to be the most accurate method of dietary exposure^14^. This paper presents the first study that explains the dietary exposure for the adult residents of HBNRA using the duplicate diet method. We investigated not only the concentration of natural radionuclides in the diets of HBNRA residents, but also artificial radionuclides and heavy metals that have received little attention in the past. This study aimed to comprehensively assess dietary exposure to radionuclides and heavy metals in HBNRA residents to provide data for local food safety regulation and epidemiological studies.

## Materials and methods

### Study areas

This study was conducted in Yangjiang city of Guangdong province, China, which is a known HBNRA and has always been the focus of radiation protection research. Yangjiang city covers an area of 7,955 square kilometers, of which hills, mountains and plains account for 26.03%, 42.73% and 22.17% respectively^17^. The fine particles of granites from nearby mountains are washed by rain into the surrounding basin regions^18^. The population is 3.02 million and most of the inhabitants lived in the study areas for many generations^17,18^. In addition, Haiyan county, which is a normal background radiation area, was selected as a control area for comparison. The county is located in the Hang-Jia-Hu Plain of the north part of Zhejiang Province. The reported average activity concentrations of ^238^U, ^232^Th and ^226^Ra in the HBNRA topsoil were 119.2 Bq/kg, 223.4 Bq/kg and 136.9 Bq/kg, respectively, which were approximately 3–4 times higher than those in the control area^19,20^.

### Food sampling and preparation

Sampling was done in HBNRA and the control area in 2018. Three households were selected at each site. It is understood that the vegetables and rice for daily consumption basically come from their own vegetable gardens and rice fields, and other foods such as meat are purchased from local shops and markets. Each household was instructed to cook ordinary everyday meals following their normal preparation habits. Participants in this survey were asked to retain the same amount of additional food consumed by two adults at each meal (excluding inedible portions and drinking water and beverages). The 5-day duplicate table-ready food of each household in HBNRA was collected as one sample, while the 3-day duplicate table-ready food of each household in the control area was collected as one sample. A total of four samples were collected from each household in March, June, August, and October 2018, respectively. The collected samples were weighed and dried at approximately 105 °C until they attained a constant weight. The water content was determined based on the sample weights after drying. They were then homogenized and sealed into Marinelli beakers for 1 month in order to allow for the secular equilibrium between the ^238^U and ^232^Th precursors and their short-lived progenies.

### Determination of radioactivity and heavy metals

^210^Pb, ^226^Ra, ^228^Ra, ^40^K and ^137^Cs were determined using a high-resolution gamma ray spectrometry from Canberra Industries. The high-purity germanium detector (BE5030) was shielded in 15 cm thick lead chamber to reduce the local radiation background. The relative efficiency of the detector was 50.5% and its energy resolution (FWHM) for full-energy peak of 1.33 MeV gamma ray of ^60^Co was 1.65 keV. GENIE-2000 software was used for the spectrometric analysis. The detector was calibrated using traceable multi-radionuclide sources from National Institute of Metrology of China. Each sample was counted for 259,200 s and similarly for background counts to provide adequate counts for the peaks of interest. Characteristic gamma ray energies were monitored to identify and quantify the radionuclides. The activities of ^210^Pb, ^40^K and ^137^Cs were directly measured on the energy peak at 46.5 keV, 1461 keV and 661.7 keV, respectively. The activities of ^228^Ra and ^226^Ra were obtained by measuring the energy peaks of their daughter products. Particularly, we decided to measure the activity of ^228^Ra using only the energy peak of ^228^Ac at 911.2 keV due to its greater photon emission probability of 26.6%. The activity of ^226^Ra was obtained by measuring the peak of ^214^Pb at 295.2 and 351.9 keV instead of ^214^Bi at 609.3 keV because of its cascade-summing effect^21^. For samples that differ significantly in density and composition from the efficiency calibration standard source, the corresponding efficiencies were constructed by LabSOCS software with characterization and traceability.

The determination of ^210^Po was conducted according to the method described by Chinese standard GB 14883.5-2016^22^. Each sample was digested in a glass beaker using HNO_3_, H_2_O_2_ and HClO_4_ with ^209^Po added as a yield tracer. Samples with high organic content should be heated repeatedly in the presence of nitric acid until the solution becomes clear. The solution was then evaporated to dryness in order to remove the HNO_3_. Before deposition, hydrazine monohydrochloride and ascorbic acid were added into the residue dissolved in HCl solution and ready for the auto-deposition of ^210^Po on a silver disc. The activity of ^210^Po was measured by alpha spectrometry. It should be noted that the activity of ^210^Po may be subject to greater uncertainty given the long delay between sampling date and measurement date^23^.

The ^129^I concentration was calculated using the measured ^127^I concentration and the ^129^I/^127^I atomic ratio. The ^127^I concentration in the sample was measured using an inductively coupled plasma mass spectrometer after extraction of iodine with alkali solution. ^129^I/^127^I atomic ratios were measured by accelerator mass spectrometry using the 3 MV Tandem AMS system at the Xi'an AMS center. The detailed procedure for the measurement of ^129^I/^127^I atomic ratio and ^127^I concentration has been described elsewhere^24,25^.

Uranium (U), Arsenic (As) and Cadmium (Cd), Mercury (Hg) and Lead (Pb) content in samples were analyzed after digestion by a mixture of concentrated HNO_3_ and H_2_O_2_. Cd, Pb and U concentrations were determined by using inductively coupled plasma mass spectrometry. As and Hg were measured with atomic fluorescence spectrometer.

### Radiological dose estimation

The radioactivity was converted into annual effective doses based on effective dose coefficients by ingestion. For adult members of the public, the recommended dose conversion coefficients for ^210^Pb, ^210^Po, ^226^Ra, ^228^Ra, ^40^K, ^137^Cs and ^129^I are 6.9 × 10^–7^, 1.2 × 10^–6^, 2.8 × 10^–7^, 6.9 × 10^–7^, 6.2 × 10^–9^, 1.3 × 10^–8^ and 1.1 × 10^–7^ Sv/Bq^26^.

The lifetime cancer risk was calculated based on the following formula as proposed by USEPA^27^:

1 $${\text{CR}}_{{\text{R}}} = {\text{ C}}_{{\text{R}}} \times {\text{I}} \times {\text{D}}_{{\text{L}}} \times {\text{f}}$$

where CR_R_ is the lifetime cancer risk, C_R_ is the activity concentration of the radionuclide in food (Bq/kg), I is the annual food intake (kg/y), D_L_ is exposure duration of life (50 years for adult), and f is the risk coefficient.

### Chemical toxicity of heavy metals

Daily intake of heavy metals (DIM) for adults was determined using the following equation^28–30^:

2 $${\text{DIM}} = {\text{C}}_{{\text{M}}} \times {\text{W}}/{\text{BW}}$$

where C_M_ is the concentration of heavy metals in food, W is the daily average consumption of food and BW is the adults body weight (60.1 kg)^31^.

Target Hazard quotient (THQ) of the chemical toxic risk was estimated according to the equation^30,32,33^:

3 $${\text{THQ}} = \left( {{\text{Efr}} \times {\text{D}}_{{\text{L}}} \times {\text{W}} \times {\text{C}}_{{\text{M}}} } \right)/ \, \left( {{\text{RfD}} \times {\text{BW}} \times {\text{AT}}} \right)$$

where Efr is the exposure frequency (365 days/year), D_L_ is the exposure duration (50 years), W is the daily average consumption of food (kg/person/day), C_M_ is the concentration of metals in food (mg/kg), RfD is the oral reference dose (mg/kg/day), BW is the adults body weight (60.1 kg), AT is the mean exposure time (365 days/year × 50 years).

## Results and discussion

### Radioactivity concentration

The activity concentrations of ^210^Pb, ^210^Po, ^226^Ra, ^228^Ra, ^40^K, ^137^Cs and ^129^I obtained in the diets of HBNRA and the control area populations are presented in Table 1. The mean activity concentrations of naturally occurring radionuclides ^210^Pb, ^210^Po, ^226^Ra, ^228^Ra and ^40^K in HBNRA were 1.0 ± 0.3, 0.7 ± 0.2, 0.8 ± 0.5, 2.2 ± 1.8 and 58 ± 25 Bq/kg dry weight (dw), respectively. In contrast, the mean activity concentration of ^210^Pb, ^210^Po, ^226^Ra, ^228^Ra and ^40^K in the control area were 1.0 ± 0.1, 0.6 ± 0.3, 0.19 ± 0.04, 0.16 ± 0.02 and 110 ± 34 Bq/kg dw, respectively. The activity concentration of ^40^K appeared to be the highest in two study areas. However, the amount of ^40^K in food is irrelevant because the body burden of ^40^K is metabolically maintained irrespective of dietary intake^34^. Furthermore, the activity concentration of ^228^Ra as the daughter of ^232^Th decay progeny was higher than that of ^238^U decay progeny ^210^Pb, ^210^Po and ^226^Ra in HBNRA. This may be due to the fact that Yangjiang is a thorium-rich region with a higher concentration of thorium than uranium in soil^19,35^.

**Table 1:** Activity concentrations of radionuclides in diet samples and the annual effective doses from ingestion in high background natural radiation area and control area.

Sample Location	Sampling time (2018)	^210^Pb (Bq/kg dw)	^210^Po (Bq/kg dw)	^226^Ra (Bq/kg dw)	^228^Ra (Bq/kg dw)	^40^K (Bq/kg dw)	^137^Cs (Bq/kg dw)	^129^I (μBq/kg dw)
High background natural radiation area	19–23 March	1.2 ± 0.2	0.5 ± 0.1	1.03 ± 0.65	2.7 ± 1.9	78.3 ± 18.1	0.07 ± 0.01	2.6 ± 0.7
	20–24 June	0.9 ± 0.5	0.8 ± 0.3	0.66 ± 0.36	2.2 ± 1.4	56.6 ± 6.9	0.07 ± 0.01	3.4 ± 1.9
	20–24 August	0.7 ± 0.4	0.7 ± 0.1	1.00 ± 0.42	2.9 ± 2.2	72.6 ± 14.6	0.06 ± 0.04	3.6 ± 1.8
	15–19 October	< 0.4	0.9 ± 0.2	0.40 ± 0.08	1.1 ± 0.4	23.2 ± 2.7	< 0.03	1.7 ± 0.2
	Mean	1.0 ± 0.3	0.7 ± 0.2	0.8 ± 0.5	2.2 ± 1.8	58 ± 25	0.06 ± 0.01	2.8 ± 1.6
	Dose (μSv/y)	55.2 ± 28.7	80.1 ± 25.2	19.8 ± 12.9	139.9 ± 112.5	32.8 ± 14.0	0.08 ± 0.01	(2.8 ± 1.6) × 10^–5^
	Cancer Risk (10^–5^)	15.3 ± 3.7	20.6 ± 6.4	4.9 ± 3.2	39.6 ± 31.4	24.5 ± 10.5	0.029 ± 0.004	(6.6 ± 3.7) × 10^–6^
Control area	17–19 March	1.1 ± 0.4	0.6 ± 0.1	0.16 ± 0.08	0.17 ± 0.09	100.4 ± 17.7	< 0.04	1.9 ± 0.5
	9–11 June	< 0.6	0.6 ± 0.2	0.15 ± 0.11	0.14 ± 0.09	85.8 ± 3.4	< 0.04	2.6 ± 1.2
	11–13 August	0.8 ± 0.4	0.7 ± 0.3	0.22 ± 0.04	< 0.2	100.8 ± 18.6	< 0.04	1.8 ± 0.6
	12–14 October	< 0.7	0.6 ± 0.4	< 0.1	< 0.2	151.7 ± 38.3	< 0.04	1.3 ± 0.2
	Mean	1.0 ± 0.1	0.6 ± 0.3	0.19 ± 0.04	0.16 ± 0.02	110 ± 34	< 0.04	1.9 ± 0.8
	Dose (μSv/y)	90.8 ± 12.2	99.4 ± 42.5	7.5 ± 1.8	14.5 ± 1.4	94.5 ± 28.9	–	(2.9 ± 1.3) × 10^–5^
	Cancer Risk (10^–5^)	20.9 ± 2.8	24.9 ± 10.4	1.8 ± 0.4	4.1 ± 0.4	68.9 ± 21.4	–	(6.7 ± 2.9) × 10^–6^

Data are presented as means ± SD.

The mean activity concentrations of artificial radionuclide ^137^Cs in HBNRA food samples was 0.06 ± 0.01 Bq/kg dw, while in the control area, the artificial radionuclide ^137^Cs activity concentrations in all of analyzed samples were below the minimum detectable activity. In addition, the mean activity concentrations of artificial radionuclide ^129^I was 2.8 ± 1.6 μBq/kg dw, which could be slightly higher than the mean activity concentration of 1.9 ± 0.8 μBq/kg dw in the food samples of the control area. This could suggest that the HBNRA may have been more affected by past human nuclear activities than the control area. However, it should be noted that the mean atomic ratio of ^129^I/^127^I in HBNRA and the control area were 1.26 × 10^–9^ and 1.15 × 10^–9^ respectively, both of which belonged to the slightly contaminated regions^10^.

The total committed effective doses due to ingestion of ^210^Pb, ^210^Po, ^226^Ra, ^228^Ra, ^40^K, ^137^Cs and ^129^I for adult were 327.9 μSv/y in HBNRA and 306.7 μSv/y in the control area. Although the effective dose in HBNRA is higher than that in the control area, there is no significant difference between them. The total effective doses were both higher than the average ingestion radiation dose of 290 μSv/y received by human being around the world^34^. However, the result is similar with the reported average value of 335.6 μSv/y in the normal background area of China^36^, and is lower than the recommended reference level of 1 mSv in a year^37^. Furthermore, the total effective dose in HBNRA in this study was lower than 610 μSv/y reported using the total diet study method^36^. Among the radionuclides evaluated, the contribution to the dose decreased as ^228^Ra > ^210^Po > ^210^Pb > ^40^K > ^226^Ra > ^137^Cs > ^129^I in HBNRA, while in the control area the order is ^210^Po > ^40^K > ^210^Pb > ^228^Ra > ^226^Ra > ^129^I > ^137^Cs. The radiation dose due to artificial radionuclides ^137^Cs and ^129^I is negligible because natural radionuclides ^210^Pb, ^210^Po, ^226^Ra, ^228^Ra, and ^40^K accounted for more than 99.9% of the total dose. The lifetime cancer risks calculated from this data were 1.1 × 10^–3^ and 1.2 × 10^–3^ for HBNRA and the control area, respectively. These values were below the reference value of 2.5 × 10^–3^ which was calculated based on the additional annual dose limit of 1 mSv for general public^38,39^. In addition, there were no difference in relation to the radiation-related cancer risk between the HBNRA and the control area.

### Heavy metal concentration

The concentration of heavy metals in diet samples in HBNRA and the control area were given in Table 2. The mean concentrations of As, Hg, Pb, Cd and U in HBNRA were found to be 0.11 ± 0.03, 0.007 ± 0.004, 3.6 ± 2.9, 0.06 ± 0.02 and 0.01 ± 0.01 mg/kg dw, respectively, whereas in the control area were 0.06 ± 0.01, 0.005 ± 0.002, 0.06 ± 0.01, 0.05 ± 0.02 and 0.003 ± 0.003 mg/kg dw. Obviously, relatively high concentrations of heavy metals, especially Pb and U, were observed in food samples from HBNRA compared to the control area. This finding is likely to be related to the local natural geology of HBNRA despite the effects of anthropogenic activities, urbanization and agricultural fertilizers.

**Table 2:** Concentrations of metals (mg/kg dw) in diet samples in high background natural radiation area and control area.

Sample location	Sampling time (2018)	As	Hg	Pb	Cd	U
High background natural radiation area	19–23 March	0.07 ± 0.01	0.006 ± 0.001	0.09 ± 0.04	0.08 ± 0.02	0.02 ± 0.01
	20–24 June	0.12 ± 0.02	0.007 ± 0.001	4.6 ± 3.2	0.05 ± 0.01	0.004 ± 0.003
	20–24 August	0.14 ± 0.01	0.006 ± 0.001	6.0 ± 2.1	0.05 ± 0.01	0.007 ± 0.006
	15–19 October	0.12 ± 0.01	0.010 ± 0.006	3.6 ± 0.4	0.06 ± 0.02	0.006 ± 0.003
	Mean	0.11 ± 0.03	0.007 ± 0.004	3.6 ± 2.9	0.06 ± 0.02	0.01 ± 0.01
Control area	17–19 March	0.06 ± 0.01	0.005 ± 0.001	–	0.04 ± 0.02	0.004 ± 0.001
	9–11 June	0.066 ± 0.004	0.006 ± 0.002	–	0.04 ± 0.03	0.0030 ± 0.0003
	11–13 August	0.07 ± 0.01	0.005 ± 0.001	0.07	0.05 ± 0.02	0.0012 ± 0.0004
	12–14 October	0.054 ± 0.004	–	0.06 ± 0.01	0.07 ± 0.00	0.005 ± 0.004
	Mean	0.06 ± 0.01	0.005 ± 0.002	0.06 ± 0.01	0.05 ± 0.02	0.003 ± 0.003

Data are presented as means ± SD.

To evaluate the health risk associated with heavy metal contamination, the daily intake of heavy metals (DIM) and target hazard quotient (THQ) for adults were calculated (Table 3). The daily intakes of As, Hg, Pb, Cd and U for adults in HBNRA were computed to be 0.47, 0.03, 15.0, 0.26 and 0.04 μg/kg bw/d, respectively, while the corresponding values for adults in the control area were 0.38, 0.03, 0.39, 0.31 and 0.02, respectively. According to the international food standard, the DIM values of Hg and Cd for both HBNRA and control area were lower than the corresponding maximum tolerable daily intake values of 0.57 and 0.83 μg/kg bw/d, respectively^40^. However, there is no clear guideline for U, Pb and As, especially for Pb and As with higher DIM values in this study.

**Table 3:** Intake and hazard quotient of metals due to ingestion in high background natural radiation area and control area.

Metals	High background natural radiation area		Control area	
	DIM (μg/kg bw/d)	THQ	DIM (μg/kg bw/d)	THQ
As	0.47 ± 0.11	1.58 ± 0.38	0.38 ± 0.06	1.28 ± 0.19
Hg	0.03 ± 0.02	0.09 ± 0.06	0.03 ± 0.01	0.07 ± 0.06
Pb	15.0 ± 12.2	3.7 ± 3.1	0.39 ± 0.07	0.03 ± 0.05
Cd	0.26 ± 0.08	2.56 ± 0.82	0.31 ± 0.14	3.15 ± 1.36
U	0.04 ± 0.04	0.18 ± 0.19	0.02 ± 0.02	0.10 ± 0.08

Data are presented as means ± SD.

THQ has been recognized as a useful parameter for assessing the risk associated with consuming food contaminated with toxic metals^33,41^. As shown in Table 3, the mean THQ values of Hg and U in both HBNRA and control area were below 1.0, implying no detrimental health effect on the consumers. However, the THQ values of As, Pb and Cd in HBNRA and the THQ values of As and Cd in the control area are all greater than 1.0, indicating that there is a potential risk associated with that heavy metal. The cumulative risk of all studied heavy metals was expressed as hazard index (HI). As shown in Table 3, it can be well observed that the HI values of HBNRA and the control area are 8.16 and 4.63, respectively, which are both higher than 1.0. Since the HI values of HBNRA is almost twice that of the control area, which means that the inhabitants around the HBNRA may have a relatively higher health risk associated with that heavy metal.

It has been reported that non-cancer mortality was higher in HBNRA among those aged less than 50 years by Zou et al.^42^, and noted that the observed increase in non-cancer mortality was unlikely to be attributable to radiation exposure, but does not give the exact reason for this finding. Given our result, the effect of heavy metals may also be a reason to explore.

## Conclusions

In this study, duplicate diet method was used to investigate the dietary exposure levels of radionuclides and heavy metals in adult residents in HBNRA. The effective doses of ingested radionuclides is 0.33 mSv/y, which is relatively higher than the global average (0.29 mSv/y), but still lower than the recommended public individual reference dose of 1 mSv/y. The result indicates that the health risk posed by the radioactivity from food intake is probably small. However, the obtained heavy metal hazard index (HI) was much higher than 1.0, which means that heavy metals may have adverse effects on human health, especially the pollution of lead, cadmium and arsenic.

As far as we know, this is by far the first detailed study of dietary exposure to radioactivity and heavy metals in HBNRA residents using duplicate diet method. The results not only provide radiation and chemical dietary exposure data for future health assessments of the study area, but also reveal a signal of the risks of chemical toxicity from ingestion to the population in HBNRA.

It must be noted that our assessment only considered radionuclides and heavy metal ingested through the diet for adult. In fact, inhalation of dust is also an important route of exposure to these substances. Furthermore, children's organs are more sensitive to radioactivity and heavy metal pollution than adults^34,43^. And it is not known whether there is an interactions between radionuclides and heavy metals. Approaches to consider the exposome should be developed to better allow consideration of effects of combined exposures^44^. For the reasons given above, further research in this area is necessary.

## Data Availability

All data generated and analyzed during this study are included in this published article.
